# Effect of propolis mouthwash on the incidence of ventilator-associated pneumonia in intensive care unit patients: a comparative randomized triple-blind clinical trial

**DOI:** 10.1186/s12903-024-04412-5

**Published:** 2024-05-29

**Authors:** Nayereh Darbanian, Monir Nobahar, Raheb Ghorbani

**Affiliations:** 1 Student Research Committee, Semnan University of Medical Sciences, Semnan, Iran; 2 Nursing Care Research Center, Semnan University of Medical Sciences, Semnan, 3513138111 Iran; 3 Department of Nursing, Faculty of Nursing and Midwifery, Semnan University of Medical Sciences, Semnan, Iran; 4 Social Determinants of Health Research Center, Semnan University of Medical Sciences, Semnan, Iran; 5 Department of Epidemiology and Biostatistics, Faculty of Medicine, Semnan University of Medical Sciences, Semnan, Iran

**Keywords:** Chlorhexidine, Clinical trial, Intensive care unit, Mouthwash, Patients, Propolis, Ventilator-associated pneumonia

## Abstract

**Objectives:**

Ventilator-associated pneumonia (VAP) increases the length of hospitalization and mortality rate. This study aimed to determine the effect of propolis mouthwash on the incidence of VAP in intensive care unit (ICU) patients.

**Materials and methods:**

Triple-blind, comparative randomized, controlled clinical trial was conducted over one year, with 110 ICU patients at Imam-Hossein and Bahar hospitals (Shahroud) and Kowsar Hospital (Semnan) in Iran. The intervention group used 15 cc of 0.06% propolis mouthwash solution twice daily at 8 AM and 4 PM for seven days. The control group used 15 cc of 0.2% chlorhexidine mouthwash at the same times and duration. Data were collected using a demographic questionnaire, APACHE II, Beck Oral Assessment Scale, and Modified Clinical Pulmonary Infection Score (MCPIS).

**Results:**

There was no significant difference in demographic information, disease severity, and oral health between the two groups before and after intervention (*P* > 0.05). The incidence of VAP in the intervention group compared to the control group was 10.9% vs. 30.9% on the third day (*P* = 0.0166, 95% CI: 0.53–0.83 and RR = 0.35), 23.6% vs. 43.6% on the fifth day (*P* = 0.0325 and 95% CI: 0.31–0.95 and RR = 0.54), and 25.5% vs. 47.3% on the seventh day (*P* = 0.0224, 95% CI: 0.32–0.92, and RR = 0.54). The Mann–Whitney indicated the incidence of VAP was significantly lower in the intervention group on the third, fifth, and seventh days.

**Conclusion:**

Propolis mouthwash can be considered as an alternative to chlorhexidine mouthwash for ICU patients.

**Clinical relevance:**

Propolis mouthwash serves as a simple, economical intervention to potentially reduce incidence of VAP.

**Trial registration:**

(IRCT20110427006318N12, date 02.04.2019).

## Introduction

Mechanical ventilation (MV) is a frequently employed therapeutic method in the intensive care unit (ICU) [1]. Ventilator-associated pneumonia (VAP) represents a serious complication associated with mechanical ventilation [2] and ranks among the most common nosocomial infections [3]. Factors such as the insertion of the endotracheal tube and duration of mechanical ventilation heighten the risk for developing VAP. The transmission of VAP occurs through the aspiration of colonized microorganisms residing in the oropharynx as well as the stomach and intestines [4]. A leading contributor to VAP is the aspiration of oral microbial colonies, often attributed to inadequate oral health care (OHC)) [5]. Studies indicate that 44–65% of patients in the ICU receive insufficient OHC [6].

The occurrence of VAP ranges from 6–52%, with a consequent mortality rate of 50–70% [7]. VAP is responsible for prolonging ICU stays and escalating mortality rates [4]. Despite significant progress in diagnosing and treating VAP in the ICU, the condition remains a prevalent source of nosocomial morbidity and mortality [8].

Pharmaceutical strategies for the prevention of VAP focus on tactics such as modulating the colonization of oral and pharyngeal bacteria, selective disinfection of the gastrointestinal tract, ulcer prevention, sedation protocols, antibiotic administration, and stringent infection control policies. Nevertheless, the prevailing consensus underscores the importance of non-pharmaceutical interventions, particularly those that prevent aspiration by effectively managing oral secretions and reducing bacterial colonization [4]. OHC constitutes a crucial element of nursing care for hospitalized patients, serving as both a preventive strategy and a cost-effective approach, especially for those in ICU. Empirical evidence underscores the significance of OHC practices for mechanically ventilated ICU patients [9]. The quality of OHC plays a pivotal role in curtailing the incidence of VAP among intubated individuals [10]. Implementing OHC protocols that include the use of mouthwash, gel, swabs, toothbrushes, or their combined application, alongside suctioning of secretions, has been shown to diminish VAP risk in this patient. Chlorhexidine-based mouthwash or gel usage can reduce VAP rates in critically ill patients from approximately 26% to near 18%, as opposed to placebo or routine care [9]. Aside from fostering oral health and aiding recovery, diligent OHC has been correlated with a decreased incidence of VAP [11]. Consequently, the employment of an effective antiseptic via OHC can lead to a lower prevalence of VAP [12].

Mouthwashes serve as an integral component of OHC [13]. Chlorhexidine mouthwash can help reduce the incidence of respiratory tract infections. Chlorhexidine is a powerful antibacterial agent used in many health products and oral medications [14]. Despite the plethora of benefits associated with chlorhexidine mouthwash, its long-term use may entail adverse effects [15]. Notable side effects comprised dysgeusia in 85.4% of users, xerostomia in 78.1%, and tooth discoloration in 58.6%. Chlorhexidine can also induce less common adverse outcomes, such as alterations in oral mucosal integrity, the onset of burning mouth syndrome (BMS), and allergic manifestations [15].

The current trend in medicine and dentistry leans towards the use of herbal products, attributed largely to their natural origin and reduced side effects [16]. Globally, the incorporation of herbal remedies as a key component of complementary and alternative medicine is expanding [17]. In the realm of oral hygiene, natural and herbal mouthwashes are emerging as favorable substitutes for synthetic products laden with chemical compounds like chlorhexidine. The natural mouthwashes reviewed encompass saltwater, baking soda, coconut oil, charcoal, propolis, seaweeds, and probiotics [18]. This shift is spurred by mounting evidence supporting the efficacy of natural substances, such as honey and propolis [13]. Numerous scientific studies over the past decades have suggested that propolis is not only effective but also safe for human use [19]. Recent research, both in-vitro and in-vivo, has offered new perspectives on the potential medicinal benefits of propolis for treating a variety of health conditions, confirming the efficacy of its bioactive components [20]. Propolis, a complex natural substance produced by bees from beeswax and plant exudates, varies in its chemical makeup based on geographical location and seasonal factors [21]. Research indicates that propolis possesses antibacterial, antifungal, and antiviral properties. It has been shown to possibly enhance the effects of antibiotics, antifungals, and antivirals, potentially reducing the required doses of these medications due to its synergistic action [19]. The clinical applications of propolis are extensive, ranging from its antioxidant, anti-inflammatory, and antimicrobial properties to antineoplastic, analgesic, and even antidepressant actions. It also shows anti-anxiety and immune-modulatory effects [20]. Propolis inhibits pathogen growth by obstructing the biological pathways required for their invasion and survival, including enzyme and protein inhibition. It disrupts cellular structures and metabolic processes vital for pathogen proliferation [22]. These multifaceted capabilities of propolis, particularly in halting cancer progression and combating an array of infections, underscore its promise as an alternative approach to bolster human health [20].

Substantial empirical evidence underscores the therapeutic efficacy of propolis in dental medicine. A comprehensive review by Abbasi et al. (2018), which examined propolis utilizations from 1997 to 2017, affirmed its effectiveness in promoting oral health, evidencing its ability to heal surgical wounds, hinder carious developments, alleviate dentine hypersensitivity, soothe aphthous ulcers, and function as a constituent in root canal irrigation solutions and mouthwashes [23]. Also, the results of a 2019 study in Tehran on the antimicrobial activity of propolis mouthwash showed that compared to chlorhexidine, propolis caused a significant difference in *Staphylococcus aureus*, *Enterococcus faecalis,* and *Lactobacillus acidophilus* [24]. The results of Eslami et al.'s (2016) study in Tabriz (Iran) on the effect of Hypozalix™ spray, propolis mouthwash, and chlorhexidine mouthwash on chemotherapy-induced mucositis in leukemia patients also showed that propolis mouthwash yields better outcomes and the patients showed a greater willingness to continue using it [25]. Results from a study indicated that chlorhexidine mouthwash was significantly more effective than the other mouthwashes such as propolis in plaque inhibition [26]. The present study aimed to determine the effect of propolis mouthwash on the incidence of VAP in ICU patients in the hospitals of Shahroud and Semnan (Iran).

## Methods

### Study design

This was a triple-blinded, randomized, controlled, clinical trial. The patients, the nurse who applied the mouthwash, the pulmonologist who diagnosed VAP, and the statistician who analyzed the data were not aware of the research groups. The study was conducted from 2022 to 2023 on 110 patients in three ICUs of Imam Hossein Hospital and three ICUs of Bahar Hospital (Shahroud), and the internal ICU of Kowsar Hospital (Semnan). The ICUs of these medical centers were similar in terms of related factors, personnel, equipment, and patients.

### Inclusion criteria

- Adult patients aged between 18 and 75 years.
- Patients mechanically ventilated (MV) for over 48 h.
- Glasgow Coma Scale (GCS) scores range from 6 to 11.
- Patients receiving enteral nutrition.
- Absence of any established OHC restrictions.
- No reported allergies to propolis or related substances.
- Obtained consent from the patient's family for participation in the study.

### Exclusion criteria

- Presence of jaw or facial trauma that complicates OHC practices.
- Confirmed immunosuppression due to treatment modalities such as chemotherapy or radiotherapy.
- Recent invasive procedures in the throat or mouth area including endoscopic interventions.
- Necessity for re-intubation during the study period.
- Presence of a tracheostomy tube, precluding standard OHC.

### Participants

The study focused on ICU patients from Imam Hossein Hospital and Bahar Hospital in Shahroud, as well as Kowsar Hospital in Semnan, who met the aforementioned inclusion criteria and did not fall under any of the exclusion categories.

### Sample size

The sample size was derived from a preliminary study that included 10 individuals in each of the two groups: the intervention group (receiving propolis mouthwash) and the control group (receiving standard care). In this initial stage, the incidence of VAP (defined by a score of 6 or more on the Modified Clinical Pulmonary Infection Score, MCPIS) was observed to be 20% within the intervention group, as opposed to 40% in the control group. To achieve a confidence level of 95% and a statistical power of 80%, a sample size calculation indicated the necessity for 55 patients per group, leading to a total of 110 participants for the clinical trial.

$$n=\frac{2\left(Z_{1-{\displaystyle\frac a2}}+Z_{1-\beta}\right)^2P\left(1-P\right)}{(P_1-P_2)}$$

### Randomization

Patients were randomly assigned to either the Control (Group A) or Intervention (Group B). The study employed blocks of four (A&B), with two individuals from each group in each block. To ensure balanced groups, patients in the opposite group were selected based on similarities in age (within ± 5 years, with a maximum difference of 10 years), sex, and mechanical ventilation mode. These variables were considered as they can influence the incidence of VAP.

### Data collection instrument

Data were collected using a demographic questionnaire, oral hygiene status was determined using Beck Oral Assessment Scale (BOAS), severity of the disease was measured based on the Acute Physiology and Chronic Health Evaluation II (APACHE II) score, and VAP diagnosis was made based on the Modified Clinical Pulmonary Infection Score (MCPIS).

### Demographic questionnaire

A questionnaire was used to record the patients' demographic information, including age, sex, underlying diseases, medications used, intubation ward, ICU referral ward, and occupation. Moreover, factors that contribute to the occurrence of VAP, including antibiotic consumption, MV mode, history of smoking and narcotic use, GCS score, the amount of support pressure, the amount of sedatives received, the use of anti-reflux medications, and gastric emptying accelerators, were recorded.

### Beck Oral Assessment Scale (BOAS)

The oral health of patients was appraised using the BOAS, encompassing five domains: lips, gums, tongue, teeth, and saliva. Each criterion is scored, culminating in a total ranging from 5 to 20. The BOAS interprets these scores as follows: a score of 5 reflects an absence of oral disorders, 6–10 signifies a mild disorder, 11–15 denotes a moderate disorder, and 16–20 represents a severe disorder [27, 28]. This instrument's validity and reliability have been confirmed internationally [17, 27]. Additionally, its reliability has been established locally by Safarabadi et al. in Iran through a test–retest method with a Pearson correlation coefficient of 0.92 [28].

### Acute Physiology and Chronic Health Evaluation II (APACHE II)

To ascertain the disease severity, the renowned APACHE II scoring system was utilized, originally proposed by Knaus et al. in 1985 [29]. The APACHE II score is a disease severity classification system and one of the most widely-used scores in the ICU [30]. This score is obtained by measuring 12 physiological variables, age, and health status. The first 12 physiological variables include body temperature, mean arterial pressure, heart rate, respiration rate, PaO2/FiO2 ratio, arterial pH, arterial HCO3, serum sodium, serum potassium, hematocrit, creatinine, and leukocytes [30] and are ranked from 0 to 4. The scoring criterion was to consider the most unusual values in the first 24 h of admission to the ICU. Consciousness level was scored based on the GCS. Scoring of the second (adjustment for age) and third (adjustment for underlying diseases) parts was performed based on specific groupings in the APACHE II form [31]. The cumulative APACHE II score characterizes the disease severity: below 16 is low; 16–25 is moderate; 26–30 is severe; and over 30 suggests very severe [32]. This tool has appropriate validity and reliability [33].

### Modified Clinical Pulmonary Infection Score (MCPIS)

VAP was diagnosed with the Modified Clinical Pulmonary Infection Score (MCPIS). This criterion is suitable for diagnosing VAP, and a score of 6 and above on this criterion indicates that the patient has VAP. This scale examines five components: Body temperature, white blood cell count, sputum, PaO2/FiO2 ratio, and chest X-ray. For each component, a score of 0 to 2 was given based on the patient's condition on the first, third, fifth, and seventh days; a score of 0 indicated normal conditions, and 1 and 2 indicated worse conditions, in respective order [34]. The reliability and validity of MCPIS have formerly been confirmed [35].

### Protocol

Throughout the study period, all conditions were identical for both the intervention and control groups. Patients were randomly assigned to either the intervention or control group and were studied for seven days.

After the patients entered the ICU, their information was recorded using the questionnaire (for demographic information), BOAS (for oral health status), APACHE II score (for disease severity), and MCPIS (for VAP diagnosis).

Propolis mouthwash solution with a concentration of 0.06% (Soren Tech Toos Pharmaceutical Company) and 0.2% chlorhexidine (Behsa Company) were prepared by the first researcher and blindly provided to the ICU nurse.

Endotracheal tube cuff pressure was measured with a gauge (Rusch, Germany) and maintained at 25 cm H2O. The conditions were the same in both groups. After washing the hands, the patient's bed was raised 30 degrees to prevent the aspiration of their secretions. After wearing clean gloves, mouthwash was applied with four to six swabs (the number of swabs used depended on the patient's oral hygiene; patients with poor oral hygiene needed more swabs) soaked in 15 cc of the 0.06% propolis solution (for the intervention group) or 15 cc of 0.2% chlorhexidine solution (for the control group), and the mucous membrane of the mouth, tongue, and gums were washed.

In the intervention group, the first mouthwash (15 cc of 0.06% propolis solution) was used by the nurse in the first 24 h of hospitalization, continuing twice per day at 8 AM and 4 PM for seven days.

The control group received an oral care regimen similar to the intervention group, with the first mouthwash (15 cc of 0.2% chlorhexidine solution) was used by the nurse in the first 24 h of hospitalization, continuing twice per day at 8 AM and 4 PM for seven days.

**Fig. 1:**
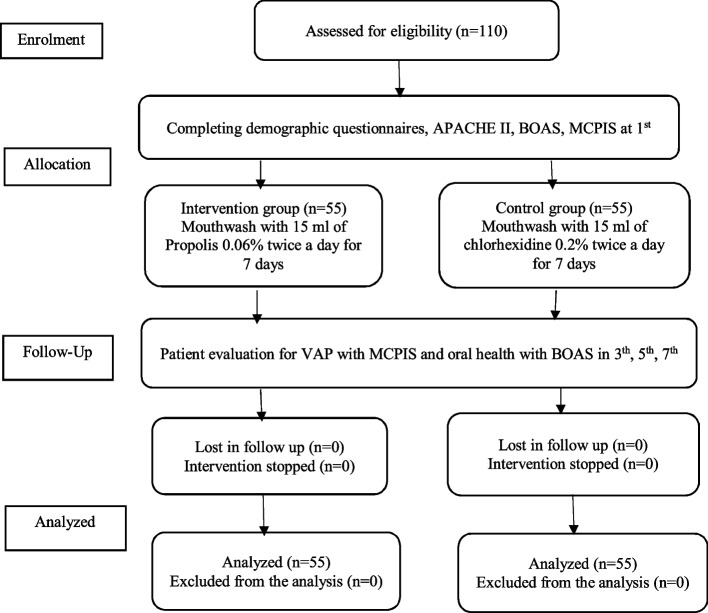
Flow chart of the study design, enrollment, allocation, randomization, follow-up and analyzed of study patients

Then, the excess secretions were suctioned using a Nelaton catheter. The suction conditions were the same for both groups. Suction tubes were changed every 24 h. Oral health condition was checked at 8 AM with BOAS, and VAP diagnosis was recorded with MCPIS on days 3, 5 (Fig. 1).

### Data analysis

The data were analyzed by Kolmogorov–Smirnov, Mann–Whitney’s U-test, Chi-square test, Fisher's exact test, and relative risk calculation at a 95% confidence interval. SPSS v. 16 was used for data recording and analysis. The significance level was 0.05.

### Ethical considerations

This research obtained a code of ethics from Semnan University of Medical Sciences (number IR.SEMUMS.REC.1397.208, date 18. 12. 2018), registered at the Iranian Registry of Clinical Trials (IRCT20110427006318N12, date 02.04.2019), and approved by Semnan University's Research Council (project number A-10–19-33). According to the CONSORT statement of the updated guidelines for reporting randomized clinical trials, preliminary to data collection, formal permissions were acquired from the relevant hospital authorities. The research purpose and procedures were explicitly communicated to the patients' primary companions, emphasizing the commitment to confidentiality and ethical management of personal information. Written informed consents were then procured from these representatives, affirming their understanding and voluntary agreement for the patients' inclusion in the study.

## Results

### Patients' demographics

Of the patients, 49.1% were male in both groups. The age of 34.5% of the patients in the intervention group and 27.3% in the control group was 70 years or above. The distribution of sex (*p* = 0.999), age (*p* = 0.341), smoking (*p* = 0.716), addiction (*p* = 0.654), APACHE II score (*p* = 0.260), employment status (*p* = 0.655), underlying disease (*p* > 0.05), medications (*p* > 0.05), ICU referral ward (*p* = 0.329), intubation ward (*p* = 0.980), GCS score (*p* = 0.341), support pressure (*p* = 0.305), and oral health status on the first, third, fifth, and seventh days (*p* > 0.05) showed no significant difference between the two groups (Table 1).

**Table 1:** Characteristics of the patients

Characteristic		Intervention (*n* = 55)		Control (*n* = 55)		*P* value
		n	%	n	%	
Gender	Male	27	49.1	27	49.1	> 0.999*
	Female	28	50.9	28	50.9	
Age	< 50	9	16.4	12	21.8	0.341**
	50–59	7	12.7	11	20.0	
	60–69	20	36.4	17	30.9	
	≥ 70	19	34.5	15	27.3	
Occupation	Employee Or worker	3	5.5	5	9.1	0.655*
	Free	10	18.2	6	10.9	
	Retired Or Unemployed	16	29.1	18	32.7	
	Others	26	47.3	26	47.3	
Underlying disease	Diabetes	14	25.5	12	21.8	0.654*
	Hypertension	17	30.9	16	29.1	0.835*
	Digestive	1	1.8	-	-	> 0.999***
	Cardiac	13	23.6	12	21.8	0.822*
	Other diseases	30	54.5	24	43.6	0.252*
Medical use	Antibiotics	53	96.4	53	96.4	-
	H_2_ blockers	2	3.6	2	3.6	-
	Antireflux	13	23.6	8	14.5	0.225*
	Antiacid	51	92.7	51	92.7	*-*
	Sedative	34	61.8	36	65.5	0.692*
Smoking	Yes	3	5.5	5	9.1	0.716***
	No	52	94.5	50	90.9	
Addiction	Yes	12	21.8	14	25.5	0.654*
	No	43	78.2	41	74.5	
APACHE II	< 15	1	1.8	2	3.6	0.260**
	15–19	10	18.2	15	27.3	
	≥ 20	44	80.0	38	69.1	
Glasgow Coma Scale	6	23	41.8	22	40.0	0.341**
	8-Jul	25	45.5	18	32.7	
	11-Sep	7	12.7	15	27.3	
BOAS	No disorder	13	23.6	7	12.7	0.768**
First day	Mild	40	72.7	48	87.3	
	Moderate	2	3.6	-	-	
Third day	No disorder	-	-	-	-	0.793**
	Mild	53	96.4	55	100	
	Moderate	2	3.6	-	-	
Fifth day	No disorder	-	-	-	-	0.417**
	Mild	53	96.4	53	96.4	
	Moderate	2	3.6	2	3.6	
Seventh day	No disorder	-	-	3	5.5	0.927**
	Mild	50	90.9	45	81.8	
	Moderate	5	9.1	7	12.7	
Referral ward to ICU	Emergency	45	81.8	45	81.8	0.329*
	Internal	-	-	2	3.6	
	Other departments	10	18.2	8	14.5	
Intubation ward	ICU	32	58.1	33	60.0	0.980*
	Emergency	20	36.4	19	34.5	
	Other department	3	5.5	3	5.5	
Support Pressure	0–5	2	3.6	-	-	0.305**
	6–10	33	60.0	43	78.2	
	11–15	20	36.4	12	21.8	

Acute Physiology and Chronic Health Evaluation II (APACHE II), Beck Oral Assessment Scale (BOAS)

*Chi-square

^**^Mann–Whitney’s U-test

^***^ Fisher's exact

### Outcome measures

*The baseline BOAS score:* on the first day (*p* = 0.768), the third day (*p* = 0.793), the fifth day (*p* = 0.417), and the seventh day (*p* = 0.917); for the lips on the first day (*p* = 0.241), the third day (*p* = 0.187), the fifth day (*p* = 0.243), and seventh day (*p* > 0.999); for the gums and oral mucosa on the first day (*p* = 0.952), the third day (*p* = 0.861), the fifth day (*p* = 0.655), and the seventh day (*p* = 0.739); for the tongue on the first day (*p* = 0.543), the third day (*p* = 0.543), the fifth day (*p* = 0.793), and the seventh day (*p* = 0.866); for the teeth on the first day (*p* = 0.703), the third day (*p* = 0.772), the fifth day (*p* = 0.342), and the seventh day (*p* = 0.564); for the saliva on the first day (*p* = 0.587), the third day (*p* = 0.573), the fifth day (*p* = 0.614), and the seventh day (*p* = 0.332); The use of the Mann–Whitney U-test across these different components of the oral health assessment indicated a consistent lack of significant difference between the two patient groups (Table 2).

**Table 2:** Characteristics of the Beck Oral Assessment Scale (BOAS) in intervention and control groups

Days	Groups	Characteristics					BOAS
		Lips	Gum	Tongue	Teeth	Saliva	
Firth	Intervention (*n* = 55)	2(1,2)	2(1,2)	1(1,1)	1(0,2)	2(1,2)	7(6,8)
	Control (*n* = 55)	2(1,2)	1(1,2)	1(1,1)	1(1,1)	2(1,2)	7(6,7)
	*P*. value*	0.241	0.952	0.543	0.703	0.587	0.768
Third	Intervention (*n* = 55)	2(2,2)	1(1,2)	1(1,1)	1(0,2)	2(2,2)	8(7,9)
	Control (*n* = 55)	2(2,2)	1(1,2)	1(1,1)	1(1,2)	2(2,2)	8(7,8)
	*P*. value*	0.187	0.861	0.543	0.772	0.573	0.793
Fifth	Intervention (*n* = 55)	2(2,2)	2(1,2)	1(1,1)	1(0,2)	3(2,3)	8(7,9)
	Control (*n* = 55)	2(2,2)	2(1,2)	1(1,1)	1(1,2)	2(2,3)	8(7,9)
	*P*. value*	0.243	0.655	0.793	0.342	0.614	0.417
Seventh	Intervention (*n* = 55)	2(1,2)	1(1,2)	1(1,1)	1(0,2)	3(3,3)	8(7,10)
	Control (*n* = 55)	2(1,2)	2(1,2)	1(1,1)	1(1,2)	3(3,3)	9(7,10)
	*P*. value*	>0.999	0.739	0.866	0.564	0.332	0.917

^*^Mann–Whitney’s U-test

*The APACHE II score*, by physiological Score (*p* = 0.293); age (*p* = 0.645) and underlying disease (*p* = 0.986) there were no significant differences between the intervention and the control groups (Table 3).

**Table 3:** Characteristics of the Acute Physiology and Chronic Health Evaluation II (APACHE II) in intervention and control groups

Characteristics	Intervention (*n* = 55)	Control (*n* = 55)	*P* value^*^
	Median (Q1, Q3)	Median (Q1, Q3)	
Physiology score	19.00(15.00,23.00)	17.00(15.00,21.00)	0.293
Age score	5.00(3.00,5.00)	3.00(2.00,5.00)	0.645
Underlying disease	0.00(0.00,1.00)	0.00(0.00,1.00)	0.986
APACHE II	23.00(20.00,27.00)	22.00(19.00,26.00)	0.260

^*^Mann–Whitney’s U-test

The baseline MCPIS score was: On the first day (*p* = 0.066), the third day (*p* = 0.002), the fifth day (*p* = 0.044), and the seventh day (*p* = 0.139); for temperature on the first day (*p* = 0.306), the third day (*p* = 0.324), the fifth day (*p* = 0.982) and the seventh day (*p* = 0.080); for the number of white blood cells on the first day (*p* = 0.286), the third day (*p* = 0.100), the fifth day (*p* = 0.009), and the seventh day (*p* = 0.035); for respiratory sputum on the first day (*p* = 0.798), the third day (*p* = 0.159), the fifth day (*p* = 0.503), and the seventh day (*p* = 0.791); for Pao_2_/Fio_2_ on the first day (*p* = 0.425), the third day (*p* = 0.282) *p*), the fifth day (*p* = 0.924), and the seventh day (*p* = 0.387); for the chest X-ray on the first day (*p* = 0.123), the third day (*p* = 0.006), the fifth day (*p* = 0.011), and the seventh day (*p* = 0.124) (Table 4).

**Table 4:** Characteristics of the Modified Clinical Pulmonary Infection Score (MCPIS) in intervention and control groups

Characteristics	Firth day		*P* value^*^	Third day		*P* value^*^	Fifth day		*P* value^*^	Seventh day		*P* value^*^
	Intervention (*n* = 55)	Control (*n* = 55)		Intervention (*n* = 55)	Control (*n* = 55)		Intervention (*n* = 55)	Control (*n* = 55)		Intervention (*n* = 55)	Control (*n* = 55)	
	Median (Q1, Q3)	Median (Q1, Q3)		Median (Q1, Q3)	Median (Q1, Q3)		Median (Q1, Q3)	Median (Q1, Q3)		Median (Q1, Q3)	Median (Q1, Q3)	
Body temperature	0(0,0)	0(0,0)	0.306	0(0,0)	0(0,0)	0.324	0(0,0)	0(0,0)	0.982	0(0,0)	0(0,0)	0.080
White blood cell count	1(0,1)	1(0,1)	0.286	1(0,1)	1(0,1)	0.100	1(0,1)	1(0,1)	0.009	1(0,1)	1(0,1)	0.035
Secretions	1(1,1)	1(1,1)	0.798	1(1,1)	1(1,1)	0.159	1(1,1)	1(1,1)	0.503	1(1,2)	1(1,2)	0.791
Pao_2_/FiO_2_ ratio	2(2,2)	2(2,2)	0.425	2(2,2)	2(2,2)	0.282	2(0,2)	2(0,2)	0.924	0(0,2)	2(0,2)	0.387
Chest X-ray	1(0,1)	1(0,1)	0.123	1(0,1)	1(1,1)	0.006	1(1,2)	2(1,2)	0.011	2(1,2)	2(1,2)	0.124
MCPIS	4(3,5)	4(4,5)	0.066	4(4,5)	5(4,6)	0.002	5(3,5)	5(4,6)	0.044	4(3,6)	5(3,7)	0.139

^*^Mann–Whitney’s U-test

The incidence of VAP in the intervention and control groups was as follows: On the third day 10.9% (*n* = 6) and 30.9% (*n* = 17), on the fifth day 23.6% (*n* = 13) and 43.6% (*n* = 24), and on the seventh day 25.5% (*n* = 14) and 47.3% (*n* = 26), respectively; the incidence of VAP was significantly lower in the intervention group on different days (Table 5).

**Table 5:** Incidence of Ventilator-associated pneumonia (VAP) in intervention and control groups

Days	VAP	Intervention (*n* = 55)		Control (*n* = 55)		Relative Risk (RR)	Confidence interval 95% relative risk	*P* value
		n	%	n	%			
Third	Yes	6	10.9	17	30.9	0.35	0.53–0.83	0.0166
	No	49	89.1	38	69.1			
Fifth	Yes	13	23.6	24	43.6	0.54	0.31–0.95	0.0325
	No	42	76.4	31	56.4			
Seventh	Yes	14	25.5	26	47.3	0.54	0.32–0.92	0.0224
	No	41	74.5	29	52.7			

There was no significant difference in VAP severity between the two groups (*p* > 0.05) (Table 6).

**Table 6:** Severity of Ventilator-associated pneumonia (VAP) in intervention and control groups

Days	Severity of VAP	Intervention		Control		*P* value
		n	%	n	%	
Third	6	5	83.3	17	100	0.562*
	7	1	16.7	-	-	
Fifth	6	11	84.6	17	70.8	0.499*
	7	2	15.4	7	29.2	
Seventh	6	4	28.6	11	42.3	0.547*
	7	8	57.1	11	42.3	
	8	1	7.1	4	15.4	
	9	1	7.1	-	-	

^*^Mann–Whitney’s U-test

## Discussion

This study aimed to determine the effect of propolis mouthwash on the incidence of VAP in ICU patients. The results showed that the incidence of VAP was significantly lower in the intervention group on the third, fifth, and seventh days, which is in line with the results of some other studies. The results of Gaber's (2020) study in Egypt also showed that propolis extract had a statistically significant effect on the prevention of VAP [36]. These findings are in line with the results of the study by Ansari Moghaddam et al. (2019) in Zahedan, Iran [37]. The study by Dehghani et al. (2019) indicated the indices that the plaque index, gingival index, and periodontal index before and after the administration of Propolis or chlorhexidine mouthwash usage did not show statistically significant differences between the two mouthwash groups [38]. The findings of the systematic review by Halboub et al. (2020) also showed in five studies, propolis, and chlorhexidine demonstrated equal efficacy in reducing plaque. Two studies favored chlorhexidine, indicating superior efficacy, while one study favored propolis. Regarding gingival inflammation outcomes, six studies were conducted, with four reporting better results with propolis, while two reported comparable outcomes between propolis and chlorhexidine [39]. Other studies have compared the effect of propolis and chlorhexidine on dental plaque and gingivitis, this study is the first to examine the effect of propolis and chlorhexidine on VAP. The results of the study by Khaky et al. (2018) in Isfahan (Iran) also revealed that the mean MCPIS score was significantly higher in the control group on the fifth day of the study than the first day, which is in line with the findings of the current study. Chlorhexidine mouthwash was used in the control group in the mentioned study and the present research, which could explain the similarity of the results [40]. The results of AkhavanKarbasi's (2016) study in Yazd, Iran, showed the anti-inflammatory role of this type of mouthwash [41]. Iftikhar et al.'s (2015) study in Pakistan also revealed that propolis mouthwash effectively reduced the incidence of VAP [42]. The results of the study by Kashi et al. (2011) are in line with the present findings [43]. Given that propolis has antibacterial and anti-inflammatory properties, propolis mouthwash reduces the incidence of VAP. The results of the study by Tavafi et al. (2020) in Tehran on the effect of propolis in vitro on some pathogenic agents revealed that propolis was less effective than chlorhexidine in inhibiting the growth of certain bacteria; still, in the present study, propolis was more effective in clinical conditions and for patients under MV [44]. Propolis mouthwash contains natural antiseptics such as pennyroyal extract, sandalwood extract, mint extract, and honey, which can reduce the number of bacteria and viruses in the mouth [42, 45]. These antiseptics can reduce the risk of respiratory infections that occur as a result of MV in hospitals. Bacteria and viruses may grow in the respiratory system of patients connected to ventilators and thus cause infection [46]. Thus, the use of propolis mouthwash can help decrease the risk of VAP in hospitals [42]. Besides, propolis is much safer than the pharmaceutical treatment of bacterial infections because it has fewer side effects than antibiotic treatments [47].

The results of this study showed that there was no significant difference in the severity of VAP between the two groups. In the study by Jahanshir et al. (2023) in Semnan [48], the severity of VAP was not significantly different between the two groups, which is consistent with this study. This consistency can be explained by the similarity of the studied subject and the method of performing OHC. The results of the study by Kord et al. (2021) in Zahedan [49] are also in line with the present findings. In the study by Khaky et al. (2018) in Isfahan [40], the severity of VAP differed significantly between the study groups; the difference between their study and ours could lie in the method of performing OHC (three times a day in the cited study and twice a day in the present study). Moreover, in their study, OHC was performed for five days, and the severity of VAP was checked on the first and fifth days, but in the current study, OHC was performed for seven days, and the severity of VAP was examined on the third, fifth, and seventh days. The results of Son et al.'s (2020) study in South Korea indicated that any intervention that can help improve oral health can play a significant role in reducing the incidence of VAP [50]. According to these results, mouthwash solutions can greatly reduce VAP by disinfecting the mouth in patients with endotracheal tubes. Patients under MV face increased morbidity and mortality due to pulmonary infection and its complications; therefore, it is necessary to identify and use pharmaceutical and non-pharmaceutical methods and antiseptic herbal products to reduce their incidence [51].

In this study, there was no significant difference in the APACHE II score between the intervention and control groups. In the study by Johnstone et al. (2021) in the US, there was no significant difference between the mean APACHE II score in the intervention and control groups [52]. In the study by Younes et al. (2022) in Egypt, more than half of the participants had an APACHE II score less than 20, which is not consistent with the results of the present study. Their study was intervention, and those with lung damage, such as pulmonary embolism, emphysema, and uncontrolled blood pressure, were not included in the study; therefore, there was a difference in the inclusion and exclusion criteria between the two studies [53].

This study showed no significant difference in BOAS between the intervention and control groups on the first, third, fifth, and seventh days. In the study by Jahanshir et al. (2023) in Semnan, there was no significant difference between the two groups in BOAS either [46]. According to the results of the study by Anggraeni et al. (2020) in Indonesia, the oral health status of intubated patients in the ICU worsened in the subscales of lips, gums, oral mucosa, and saliva despite the regular administration of chlorhexidine gluconate; this result is not consistent with this study. In the cited study, the effect of chlorhexidine was investigated alone, and the goal was not to investigate the effect of OHC on the incidence of VAP [31].

In the present study, bronchoalveolar lavage, which is a reliable and definitive method for diagnosing VAP, was not used due to its invasiveness. Several factors, such as different immune systems, antibiotic resistance, and the presence of resistant pathogens in the environment, can contribute to VAP incidence, which could not be controlled. Also, it did not investigate different types of VAP based on the causative pathogen, nor did it explore various oral and dental care regimens. Additionally, this study has limitations concerning infants, children, and elderly patients. Future studies should examine the effect of propolis mouthwash on the incidence of VAP in non-intubated patients. Besides, the effect of propolis mouthwash on the incidence of VAP should be investigated in children.

## Conclusion

According to the present study, propolis mouthwash is effective in reducing the risk of VAP. Propolis mouthwash can be used as an alternative to chlorhexidine for MV patients in the ICU.

## Data Availability

The datasets used and/or analysed during the current study available from the corresponding author on reasonable request.
